# A case series of sage: a new couple-based intervention for borderline personality disorder

**DOI:** 10.1186/s40479-023-00244-x

**Published:** 2024-01-12

**Authors:** Skye Fitzpatrick, Sonya Varma, David Chafe, Nikoo Norouzian, Jenna Traynor, Sophie Goss, Elizabeth Earle, Alyssa Di Bartolomeo, Ashley Siegel, Lindsay Fulham, Candice M. Monson, Rachel E. Liebman

**Affiliations:** 1 Department of Psychology, York University, 4700 Keele Street, Toronto, ON M3J 1P3 Canada; 2 Department of Psychology, Toronto Metropolitan University, Toronto, ON Canada; 3 Department of Psychiatry, University Health Network, University of Toronto, Toronto, ON Canada; 4 McLean Hospital, Harvard Medical School, Boston, MA USA

**Keywords:** Borderline personality disorder, Emotion dysregulation, Suicide, Self-injury, Couple therapy, Intervention development

## Abstract

**Background:**

Research suggests that interpersonal dysfunction may be central to borderline personality disorder (BPD), and that the relationships of people with BPD are particularly impaired. Further, the significant others of people with BPD exhibit elevated psychological problems but little access to mental healthcare. Despite this, most BPD interventions are delivered individually and do not routinely incorporate significant others. This manuscript presents the first case series of Sage, a 12-session manualized intervention for people with borderline personality disorder (BPD) and their intimate partners with three targets: a) BPD severity, b) relationship conflict, and c) intimate partner mental health.

**Findings:**

Five couples of people with BPD with frequent suicidal/self-injurious behavior or high suicidal ideation and their intimate partners received Sage. Measures of Sage targets as well as tertiary outcomes were administered at pre-, mid-, and post-intervention. Four out of five dyads completed Sage, with high intervention satisfaction ratings. Improvements were generally demonstrated in BPD severity, suicidal ideation, and suicidal behavior/self-injury. Half of dyads exhibited improvements in conflict, and additional improvements in mental health outcomes for dyad members were demonstrated. One dyad exhibited poor outcomes and speculations regarding this are offered.

**Conclusions:**

Findings provide proof of concept of Sage as an intervention that can improve BPD and other mental health outcomes in those with BPD and their intimate partners. Incorporating intimate partners into BPD treatment may optimize and expedite its outcomes. However, further testing is needed.

**Trial registration:**

This project was pre-registered at Clinicaltrials.gov (Identifier: [NCT04737252]).

People with borderline personality disorder’s (BPD’s) intimate relationships are associated with dysfunction, communication problems, and dissatisfaction [1, 2]. The Borderline Interpersonal-Affective Systems (BIAS) model suggests that BPD is maintained through transactions between people with BPD’s and their significant others’ (SOs) dysregulated emotions and communication, and SOs may also inadvertently reinforce destructive behavior in BPD. Including SOs in treatment may therefore optimize BPD interventions by targeting each member’s cognitive, emotional, and communication processes, and the transaction between them. Conjoint interventions may also target SOs’ mental health problems [1].

No manualized interventions target BPD, relationship problems, and SO mental health simultaneously. As a result, our team developed Sage [3], a manualized psychotherapy delivered conjointly to people with BPD and SOs to target BPD, relationship functioning, and SO mental health. Sage is outlined in detail elsewhere [3] but, in brief, is a 12-session intervention that targets the relational and emotional maintenance factors of BPD outlined by the BIAS model [4]. Phase 1 provides BPD psychoeducation and skills to mitigate safety concerns and relationship conflict. For example, these skills include learning how to monitor oneself for signs of escalating distress; effectively disengaging from the conflict (i.e., calling a “time out”); using strategies to decrease distress (e.g., paced breathing); and returning to the conversation when emotions are regulated and key areas of focus for the conflict are refined. Phase 2 teaches dyadic emotion regulation and effective communication skills. Phase 3 focuses on cognitions that influence emotion dysregulation and relationship dysfunction, dyadic strategies to challenge them, and relapse prevention. Most sessions are 75-min and weekly, although the first two sessions are 90-min within the same week where possible to support safety planning.

This manuscript describes a proof-of-concept case series of five individuals with BPD and their partners who received Sage from study investigators or supervised doctoral-level clinical psychology students. The purpose of this case series was to gather preliminary evidence regarding whether Sage is acceptable and can improve BPD symptoms (primary outcome), relationship conflict and SO mental health (secondary outcomes), and other relevant tertiary outcomes.

## Method

### Participants

Five adult intimate dyads participated, wherein one partner (1) met DSM-5 BPD criteria [5]; and (2) had elevated suicidal ideation (≥ 15 on the Beck Scale for Suicidal Ideation [6]) or chronic and recent suicidal or non-suicidal self-injury (2 + acts in the past five years with 1 + in the past eight weeks; e.g., [7]). Exclusion criteria included (1) severe, past-year intimate partner violence; (2) lack of English proficiency; (3) residing outside Ontario; and (4) clinically-significant psychosis not explained by BPD; bipolar I disorder with past-month mania or a past-year hospitalization for mania; severe current substance use disorder; or major cognitive, intellectual, and/or medical impairment.

### Measures

All measures are described in Table 1. BPD, exclusion criteria, and comorbidities were assessed with diagnostic interviews. Assessors were calibrated quarterly against a gold-standard rater. Primary outcomes were participants with BPD’s BPD symptoms (self- and partner informant-report), suicidal/self-injurious behavior, and suicidal ideation (self- and partner informant-report). Secondary outcomes involved self-reports of conflict from both members, and partners’ reports of their emotion dysregulation, shame, depression, anxiety, positive affect, negative affect, and anger/hostility. Participants with BPD also provided informant-ratings of partners’ emotion dysregulation. Tertiary outcomes included participants with BPD’s self-reports of secondary outcomes, partners’ informant-report of their emotion dysregulation, and both members’ self-report of relationship satisfaction and intervention satisfaction. This case series was pre-registered (Clinicaltrials.gov Identifier: NCT04737252).

**Table 1:** Domains assessed, measures used, and measure citations

Domain assessed	Measure		Citation		Type of measure	
**Diagnostic and screening measures**						
BPD	*The International Personality Disorders Examination-BPD Module*		[8]		Interview completed by both members of couple	
Psychosis, mania and/or hypomania, substance and alcohol abuse, comorbid psychological disorders	*The Diagnostic Assessment Research Tool*		[9]		Interview completed by both members of couple	
Lifetime frequency and lethality of past suicidal and self-injurious behavior	*The Lifetime–Suicide Attempt Self-Injury Count*		[10]		Interview completed by people with BPD	
**Primary outcomes: BPD symptoms**						
BPD symptoms	*Borderline Symptom List-23*		[11]		Self-report completed by people with BPD	
BPD symptoms (informant-report)	*Borderline Symptom List-230 informant report*		[11]		Informant-report completed by partners	
Frequency of suicidal/self-injurious behavior in the past four weeks	*Suicide Attempt Self-Injury Interview*		[12]		Interview completed by people with BPD	
Suicidal ideation	*Beck Scale for Suicidal Ideation*		[6]		Self-report completed by people with BPD	
Suicidal ideation (informant-report)	*Beck Scale for Suicidal Ideation*		[6]		Informant-report completed by partners	
**Secondary and tertiary outcomes: Partner mental health and conflict**						
Conflict	*Ineffective Arguing Inventory*		[13]		Self-report completed both members of couple	
Emotion dysregulation	*Difficulties in Emotion Regulation Scale*		[14]		Self-report completed by partners	
Emotion dysregulation (informant report)	*Difficulties in Emotion Regulation Scale – Partner Version*		[15]		Informant-report completed by people with BPD	
Shame	*The Experience of Shame Scale*		[16]		Self-report completed by partners	
Depression	*The Patient Health Questionnaire*		[17]		Self-report completed by partners	
Anxiety	*The Generalized Anxiety Disorder-7*		[18]		Self-report completed by partners	
Positive affect, negative affect, and anger/hostility	*Positive and Negative Affect Schedule-X subscales*		[19]		Self-report completed by partners	
**Tertiary Outcomes: Individual Mental Health and Intervention Satisfaction**						
People with BPD completed the above self-report measures of emotion dysregulation (with partners completing informant-reports), shame, depression, anxiety, positive affect, negative affect, and anger/hostility as tertiary outcomes						
Relationship satisfaction		*Couples Satisfaction Inventory*		[20]		Self-report completed by both members of couple
Intervention satisfaction		*The Client Satisfaction Questionnaire-8*		[21]		Self-report completed by both members of couple

*BPD* Borderline personality disorder

### Procedures

Study procedures received research ethics approval. After an online screening, prospective participants with BPD and SOs completed eligibility assessments. Couples were administered outcome assessments at baseline, after session 6,^1^ and at the end of Sage.

Sage therapists who were not intervention developers learned the intervention from reading the manual and watching and discussing Sage intervention session recordings. Graduate students conducted co-therapy for their first case (e.g., two student therapists present instead of one; this occurred in one instance in the case series). Sage therapists met with study investigators for weekly group supervision which included review of session recordings.

### Data analytic strategy

Jacobson and Truax’s [22] Reliable Change (RC) indices were calculated for each outcome to classify responses as improved, worsened, or unchanged.^2^ Reliability and standard deviation estimates used to calculate RC for each measure were obtained from studies using samples of people with BPD or related problems (e.g., inpatient psychiatric samples).

## Results

See Table 2 for sample demographic and clinical characteristics. See Table 3 for means, standard deviations, and RC thresholds for each outcome. See Table 4 for reliable improvement, worsening, or no change outcomes for each measure by couple. Four of five couples completed the intervention, and one dropped out of the intervention after session 10 without providing post-intervention data. This couple is excluded from RC analyses. Primary and secondary outcomes are described for each case below.

**Table 2:** Demographic and current comorbid information

	**Participants with BPD**	**Partners**
**Most prevalent demographics in the sample**		
Mean age (SD)	25.40 (4.04)	25.60 (2.07)
Majority gender	Cis female (80%)	Cis male (100%)
Majority sexual orientation	Heterosexual (60%)	Heterosexual (80%)
Majority race/ethnicity	White (100%)	White (100%)
Majority marital status	Never married (100%)	Never married (100%)
Mean relationship length of couples (SD)	1.81 years (1.76)	
Number of couples currently cohabitating	*n* = 4 (80%)	
**Current comorbidities**		
Current mood disorder	*n* = 4 (80%)	*n* = 1 (20%)
Current anxiety disorder or obsessive–compulsive disorder	*n* = 5 (100%)	*n* = 3 (60%)
Current posttraumatic stress disorder	*n* = 4 (80%)	*n* = 0 (0%)
Current substance use disorder	*n* = 2 (40%)	*n* = 1 (20%)

*SD* Standard deviation, *BPD* Borderline personality disorder

**Table 3:** Pre, mid-, and post-intervention means and standard deviations for study measures for people with BPD and their partners

		**People with BPD**				**Partners**			
**Construct**	**Measure Range**	**Pre-Sage** **M(SD)**	**Mid-Sage** **M(SD)**	**Post-Sage** **M(SD)**	**RC Index** (Sdiff × 1.96)	**Pre-Sage** **M(SD)**	**Mid-Sage** **M(SD)**	**Post-Sage** **M(SD)**	**RC Index** (Sdiff × 1.96)
BPD severity	0–4	2.90(.72)	2.81(.45)	1.77(.98)	.43	**-**	**-**	**-**	**-**
BPD severity (informant-report)	0–4	2.49(1.02)	1.75(.76)	1.71(.57)	.43	**-**	**-**	**-**	**-**
Suicidal ideation	0–38	17.60(2.61)	14.00(4.64)	6.25(12.50)	6.74	**-**	**-**	**-**	**-**
Suicidal ideation (informant report)	0–38	10.80(7.26)	7.20(6.54)	.00(.00)	6.74	**-**	**-**	**-**	**-**
Lifetime suicide attempt frequency	0 and up Range in sample: 4–175	47.60(71.97)	**-**	**-**	**-**	**-**	**-**	**-**	**-**
Highest lethality of lifetime suicidal/self-injurious act	1–6	5.80(.45)	**-**	**-**	**-**	**-**	**-**	**-**	**-**
Suicide/self-injury episodes^a^	0 and up	2.8(4.66)	3.2(5.02)	1.25(1.5)	**-**	**-**	**-**	**-**	**-**
Conflict	0–90	18.60 (6.73)	18.40(4.56)	19.00(10.89)	2.73	19.20(2.39)	19.40(4.72)	19.50 (4.51)	2.53
Emotion Dysregulation	36–180	117.00(26.68)	117.60(25.44)	95.25(29.04)	13.85	93.80(35.12)	93.60(25.31)	96.25(20.92)	13.85
Emotion Dysregulation (informant-report)	5–40	26.60(6.80)	27.80(6.14)	25.75(5.44)	5.10	20.20(8.47)	14.00(2.00)	15.50(1.29)	5.34
Depression	0–27	20.20(7.79)	19.80(5.81)	14.75(7.72)	7.19	11.80(7.19)	11.80(3.27)	13.75(2.87)	7.19
Anxiety	0–21	16.40(5.55)	15.20(3.63)	11.50(4.20)	5.08	7.00(5.15)	8.20(4.97)	7.00(3.83)	5.08
Shame	39–195	83.60(15.79)	86.00(15.84)	73.25(6.99)	11.49	57.60(14.99)	56.60(20.37)	63.00(13.19)	11.49
Emotional Reactivity	0–84	75.20(5.72)	66.80(11.30)	65.50(7.14)	11.90	28.00(21.46)	25.60(20.86)	26.75(19.09)	11.90
Positive Emotion	10–50	19.40 (7.44)	18.40 (7.70)	23.75 (6.90)	7.20	27.80 (5.45)	27.00 (7.04)	26.25 (7.63)	7.20
Negative Emotion	10–50	36.60 (7.77)	33.80 (6.34)	29.25 (9.98)	6.20	23.40 (11.59)	22.80 (7.86)	20.50 (5.92)	6.20
Anger	6–35	14.40 (5.37)	15.60 (5.68)	13.25 (5.47)	6.10	11.60 (6.19)	12.40 (5.22)	12.50 (3.70)	6.10
Relationship Satisfaction	0–160	132.00(16.06)	122.20(24.18)	109.00(53.52)	12.04	126.80(24.01)	120.60(23.50)	112.25(27.10)	12.57

*BPD *Borderline personality disorder, *M* Mean, *SD* Standard Deviation, *RC* Reliable change;—= not assessed; ^a^Number of episodes in the past 4 weeks (reliable change not calculated)

**Table 4:** Reliable improvement, worsening, or no change results by dyad

	BPD severity	BPD severity (informant-report)	Suicidal ideation	Suicidal ideation (informant- report)	Conflict	Emotion Dysregulation	Emotion Dysregulation (informant-report)	Depression	Anxiety	Shame	Emotion Reactivity	Positive Emotion	Negative Emotion	Anger	Relationship Satisfaction
**Case 1**															
BPD +	*✓*	*✓*	*✓*	*✓*	*✓*	*✓*	*✓*	NC	*✓*	NC	*✓*	NC	NC	NC	*✓*
Partner					*✓*	NC	*✓*	X	NC	X	NC	X	NC	NC	NC
**Case 2**															
BPD +	*✓*	*✓*	*✓*	*✓*	*✓*	*✓*	NC	*✓*	*✓*	*✓*	NC	*✓*	*✓*	*✓*	NC
Partner					*✓*	*✓*	*✓*	NC	NC	NC	*✓*	NC	*✓*	NC	NC
**Case 3**															
BPD +	*✓*	*✓*	*✓*	*✓*	NC	NC	NC	NC	NC	*✓*	*✓*	X	NC	NC	X
Partner					NC	*✓*	NC	NC	NC	NC	NC	*✓*	NC	NC	X
**Case 4**															
BPD +	X	NC	X	NC	X	X	NC	NC	NC	NC	NC	NC	NC	NC	X
Partner					X	X	NC	NC	NC	X	NC	NC	NC	NC	X

Per Jacobson and Truax [22], absolute change values above Sdiff × 1.96 constitute reliable change

*✓* = reliable improvements; X = reliable worsening; *NC* No reliable change; - = not assessed; *BPD* Borderline personality disorder, *BPD +*  Participants with borderline personality disorder

Participant with BPD #1 exhibited pre- to post-Sage *improvement* in BPD severity, suicidal ideation, conflict, emotion dysregulation, anxiety, emotional reactivity, and relationship satisfaction. They exhibited *no change* in depression, shame, positive emotion, negative emotion, and anger. Partner #1 exhibited *improvement* in conflict and informant-reported emotion dysregulation, *worsening* in depression, shame, and positive emotion, and *no change* in self-reported emotion dysregulation, anxiety, emotional reactivity, negative emotion, anger, and relationship satisfaction.

Participant with BPD #2 exhibited pre- to post-Sage *improvement* in BPD severity, suicidal ideation, conflict, self-reported emotion dysregulation, depression, anxiety, shame, positive emotion, negative emotion, and anger. They exhibited *no change* in informant-reported emotion dysregulation, emotional reactivity, or relationship satisfaction. Partner #2 exhibited *improvement* in conflict, emotion dysregulation, emotional reactivity, and negative emotion, and *no change* in depression, anxiety, shame, positive emotion, anger, and relationship satisfaction.

Participant with BPD #3 showed *improvement* in BPD severity, suicidal ideation, shame, and emotional reactivity from pre- to post-Sage. They exhibited *no change* in conflict, emotion dysregulation, depression, anxiety, negative emotion, and anger, and *worsening* in positive emotion and relationship satisfaction. Partner #3 exhibited *improvement* in self-reported emotion dysregulation and positive emotion, *no change* in conflict, informant-reported emotion dysregulation, depression, anxiety, shame, emotional reactivity, negative emotion, and anger, and *worsening* in relationship satisfaction.

Participant with BPD #4 exhibited *no change* in informant-reported BPD severity, suicidal ideation, and emotion dysregulation, but they self-reported *worsening* in each of these domains along with conflict and relationship satisfaction. Finally, they exhibited *no change* in depression, anxiety, shame, emotional reactivity, positive emotion, negative emotion, and anger. Partner #4 exhibited *worsening* in conflict, self-reported emotion dysregulation, shame, and relationship satisfaction, and *no change* in informant-reported emotion dysregulation, depression, anxiety, emotional reactivity, and positive emotion, negative emotion, and anger.

Across couples, intervention satisfaction was high for participants with BPD and partners (Mean = 29.75 out of 32 for both; *SD* = 2.87 and 2.50, respectively). RC in the frequency of suicidal/self-injurious acts in the past month was not computed because this index does not have Cronbach alphas. However, on average across the four participants with post-intervention data, the frequency of suicidal and self-injurious behaviors in the past-month decreased from baseline (Mean = 3.25, *SD* = 5.25) to post-Sage (Mean = 1.25; *SD* = 1.5).

## Discussion

Results provide preliminary evidence that Sage is a promising brief conjoint intervention for participants with BPD and partners. Couples found the intervention highly acceptable, with four out of five completing it and the fifth coming close to completion. Three of four participants with BPD and their partners agreed that there were improvements in BPD severity and suicidal ideation, and the average frequency of suicidal/self-injurious behaviors across participants showed a reduction from pre- to post-Sage. Moreover, three of four participants with BPD improved in other mental health symptoms.

Although three of four couples exhibited largely positive outcomes, one couple exhibited no change or poor outcomes which accounted for almost all instances of worsening. Sage may not have been beneficial or possibly even iatrogenic for this couple. This couple may have experienced a considerable stressor during the post-assessment period, resulting in the post-assessment capturing acute but temporary relational distress. Alternatively, Sage may have raised awareness of significant relationship issues for this couple, increasing their post-assessment distress. Further testing is needed to understand who may and may not be good candidates for Sage, or whether these outcomes would be sustained at a follow-up.

Secondary and tertiary outcomes were generally positive but less consistent than primary outcomes. Conflict improved in half of the couples, did not change in one couple, and worsened in the couple discussed above. Similarly, half of partners reported improvements in mental health outcomes. Ceiling and floor effects may explain the more limited improvement in these domains. Average relationship satisfaction remained well above the clinical threshold (*M* = 104; [20]) across all assessments, and partner baseline mental health problems were relatively low, which may have limited the detection of change. However, it is also possible that the benefits of partner involvement in Sage are specific to BPD outcomes.

We are unable to identify meaningful patterns in outcomes in the absence of a control group, a larger sample, and follow up assessment. Sample diversity was also limited mainly to white, heterosexual couples with female-identifying participants with BPD who were, on average, young and early in their relationship. Greater demographic variability is needed in future work. However, our preliminary findings are encouraging and provide proof-of-concept that Sage may have a positive impact on symptoms of BPD as well as some partner and relationship outcomes.

## Data Availability

The data utilized in this project are not available due to privacy concerns related to the size of the sample.

## Footnotes

1 One participant with BPD completed their mid-assessment after session 7, rather than session 6.

2 Jacobson and Truax (1991) formula for RC: $\frac{{x}^{1}-{x}^{2}}{{\text{Sdiff}}}$ wherein Sdiff= $\sqrt{{2({{\text{S}}}_{{\text{E}}})}^{2}}$ and ${{\text{S}}}_{{\text{E}}}$=${S}_{1}\sqrt{1-{r}_{xx}}$
